# Global, regional and national burden, incidence, and mortality of cervical cancer

**DOI:** 10.1002/cnr2.1756

**Published:** 2022-12-21

**Authors:** Zohre Momenimovahed, Afrooz Mazidimoradi, Parang Maroofi, Leila Allahqoli, Hamid Salehiniya, Ibrahim Alkatout

**Affiliations:** ^1^ Qom University of Medical Sciences Qom Iran; ^2^ Shiraz University of Medical Sciences Shiraz Iran; ^3^ Department of Epidemiology, School of Public Health Hamadan University of Medical Science Hamadan Iran; ^4^ Iran University of Medical Sciences Tehran Iran; ^5^ Social Determinants of Health Research Center Birjand University of Medical Sciences Birjand Iran; ^6^ Campus Kiel, Kiel School of Gynecological Endoscopy University Hospitals Schleswig‐Holstein Kiel Germany

**Keywords:** burden, cervical cancer, global, incidence, mortality

## Abstract

**Aim:**

Among gynecological cancers, cervical cancer is the most common cause of cancer‐related death in developing countries. This study analyzes the incidence, mortality, and burden of cervical cancer using the Global Burden of Disease (GBD) 2019 study.

**Materials and Methods:**

The GBD (2019) data on cervical cancer was extracted from the Global Health Data Exchange (GHDx) query tool. Age‐standardized rate (ASR) incidence, deaths, lost years of life (YLLs), years of life with disabilities (YLDs), and adjusted years of life with disabilities (DALYs) of cervical cancer in women were extracted. Data were extracted globally for 204 countries and groups based on a socio‐demographic index (SDI), World Health Organization (WHO) regions, continents, World Bank regions, and 22 GBD regions.

**Results:**

The higher standardized age incidence of cervical cancer is in lower SDI countries, Africa, the African region (According to the WHO), and Sub‐Saharan Africa (According to GBD regions). The highest deaths of ASR is in countries with low SDI, low‐income group, Africa, the African region (According to the World Health Organization), and Sub‐Saharan Africa (According to GBD regions). According to SDI classification, the highest DALYs ASR is in low SDI countries, World Bank Low‐income countries, African and then American continents, African region, Sub‐Saharan Africa, and then Latin America & Caribbean‐WB (Based on GBD regions).

**Conclusion:**

In 2019, incidence, mortality, and DALYs of cervical cancer mostly affected countries with lower socioeconomic status. Given that cervical cancer is highly preventable, access to screening services and the presence of trained and knowledgeable health care staff can reduce illness, suffering, and death caused by this malignancy. It is recommended to use the national and international potentials to reduce the incidence of this malignancy.

## 1 INTRODUCTION

After heart disease, cancer is the second most common cause of death in the world, which imposes a significant burden on healthcare systems.^1^ Among gynecological cancers, cervical cancer is the most common cause of cancer‐related death in countries with low and middle Human Development Index (HDI) countries.^2^ Cervical cancer has the highest incidence among young women.^3^ Adenocarcinoma and squamous cell carcinoma are the two most common histological types of cervical cancer.^4^ Infection with human papillomavirus carcinogens is a necessary cause of invasive cervical cancer. The progression of intraepithelial dysplastic lesions following persistent HPV infection leads to cervical cancer.^5^ HPV is a sexually transmitted virus, so factors such as young age at marriage, multiple sexual partners, and unprotected sex, which increase the risk of contracting this virus, are linked with cervical cancer. Although HPV is a necessary cause of cervical cancer, not all lesions lead to malignancy. Some cofactors, such as multiparity, young age at marriage, and the use of oral contraceptives, in combination with HPV, cause malignant deformities of cancer cells.^6^

Knowing that human papillomavirus carcinogenesis is the leading cause of cervical cancer has opened up new avenues for prevention. Also, early detection and treatment of precancerous lesions have prevented a significant proportion of morbidity and mortality associated with cervical cancer.^3^ Although there has been a gradual decline in cancer‐related incidence and mortality, cervical cancer still imposes a significant burden on health systems. Decreased screening coverage, poor screening tests to identify precancerous lesions, and lack or inadequate treatment are responsible for most of the cost of cervical cancer in some societies.^7^ Unfortunately, 80% of women in developing countries attend medical centers after the onset of symptoms.^8^ These factors along with cultural and socio‐economic factors, as well as societies' norms explain some of the geographical differences in the incidence of cervical cancer in different parts of the world.^7^ According to statistics, cervical cancer is the most common disease in low‐income areas, with more than two‐thirds of cases and deaths occurring in developing countries.^9^

At present, the development of effective prevention programs requires accurate statistics. Statistics, in addition to showing the current situation, make it necessary to take action to improve the situation, allocate the necessary budget and facilities, make purposeful planning, and finally select the best possible approach.^10^ Therefore, the present study has gathered and presented epidemiological data, including incidence cases, age‐standardized incidence rate, deaths, age‐standardized mortality rate, YLLs, YLDs, and DALYs from the study of GBD in 2019.

## 2 MATERIALS AND METHODS

In summary, the GBD (2019) data on cervical cancer was extracted from the GHDx query tool. GBD (2019) has systematically and comprehensively estimated 286 causes of death, 369 causes of diseases and injuries, and 87 risk factors for 204 countries and regions. Geographically, the GBD divides the world into 7 main regions and 21 subregions. Detailed information on the data sources used in the present study can be found at GBD 2019 (http://ghdx.healthdata.org/gbd-2019/data-input-sources).

In this study, the variables obtained from GBD statistics include cervical cancer incidence, mortality rate, years of life lost due to premature mortality (YLLs), years lived with disability (YLDs), disability‐adjusted life years (DALYs), age‐standardized incidence, and mortality rates per 100 000 people in 2019 based on socio‐demographic Index (SDI) indicators. These variables also included World Bank income levels, continents, WHO regions, and GBD regions. The Socio‐demographic Index (SDI) is a composite indicator of a country's lag‐distributed income per capita, average years of schooling, and the fertility rate in females under the age of 25 years.^11^ Overall, SDI = 0 has the lowest level of health‐related development and SDI = 1 has the highest health‐related development. In the GBD study (2019), countries and territories were classified as low, low‐medium, medium, medium‐high, and high based on the SDI.^12^

In this study, a description of the mentioned indicators was done separately for each group by crude and age‐standardized rates. Age‐standardized mortality rates can be used to compare national mortality rates without being affected by differences in age distribution across countries. Without such standardization, it would be difficult to determine if the different mortality rates are due to age or other factors.^13^ For GBD, an internationally standardized form of QALY has been developed, known as the Adjusted Year of Life (DALY). DALY is defined as the years of life lost due to premature death and the years lived with a disability of specified severity and duration. A DALY is therefore a wasted year of healthy living. “Premature” death is defined as a death occurring before the age at which the dying person would have expected to survive if they were part of a standardized population with a life expectancy at birth equal to that of the longest surviving population in the world, Japan. For calculating the total number of DALYs for a given condition in a population, years of life lost (YLLs) and years of disability of known severity and duration (YLDs) for this condition should be estimated and then added together.^14^ More details presented in Table 1.

**TABLE 1:** Summary of comparison indicators

Indicator	Definition	Formula/ components
disability‐adjusted life years (DALYs)	One DALY is the equivalent of losing 1 year of full health.^15^ Health interventions are designed to prevent DALY and, in doing so, increase the number of healthy years of life.^15^	DALYs = YLDs + YLLs.^15^
Years of life lost (YLLs)	Years of life are lost as a consequence of premature mortality^16^; Were estimated by multiplying the estimated number of deaths by the normal life expectancy for the respective age^17^ and Measures the life expectancy reduction.^18^	$\text{YLLs}\left[r,K,\beta \right]=\frac{\mathrm{KC}e^{\mathrm{ra}}}{{\left(r+\beta \right)}^{2}}\left\{e^{-\left(r+\beta \right)\left(l+a\right)}\left[-\left(r+\beta \right)\left(L+a\right)-1\right]-e^{-\left(r+\beta \right)a}\left[-\left(r+\beta \right)a-1\right]\right\}+\frac{1-K}{r}\left(1-e^{-\mathrm{rL}}\right)$ *K* = age weighting modulation factor; *C* = constant; *r* = discount rate; *a* = age of death; β = parameter from the age weighting function; *L* = standard expectation of life at age *a*.^18^
Years lived with disability (YLDs)	One YLD represents the equivalent of one full year of healthy life lost due to disability or ill‐health^15^ and A measure of years of life without perfect health.^19^	$\text{YLDs}\left[r,K,\beta \right]=D\left\{\frac{\mathrm{KC}e^{\mathrm{ra}}}{{\left(r+\beta \right)}^{2}}\left\{e^{-\left(r+\beta \right)\left(l+a\right)}\left[-\left(r+\beta \right)\left(L+a\right)-1\right]-e^{-\left(r+\beta \right)a}\left[-\left(r+\beta \right)a-1\right]\right\}+\frac{1-K}{r}\left(1-e^{-\mathrm{rL}}\right)\right\}$ *K* = age weighting modulation factor; *C* = constant; *r* = discount rate; *a* = age of death; β = parameter from the age weighting function; *L* = duration of disability; D = disability weight.^18^
World Bank regions	Classifies economies for analytical purposes into four income groups: low, lower‐middle, upper‐middle, and high income.^20^	uses gross national income (GNI) per capita data in U.S. dollars, converted from local currency using the World Bank Atlas method, which is applied to smooth exchange rate fluctuations.^20^
Socio‐demographic Index (SDI)	Is a summary indicator to represent background levels of social and economic conditions that can influence health outcomes in a given location.^21^	A composite indicator (geometric mean) of a country's lag‐distributed income per capita, average years of schooling of ages 15 and older, and the fertility rate in females under the age of 25 years.^21^ SDI = 0 has the lowest level of health‐related development and SDI = 1 has the highest health‐related development. Detailed classification are low SDI (<0·45), low‐middle SDI (≥0·45 and <0·61), middle SDI (≥0·61 and <0·69), high‐middle SDI (≥0·69 and <0·80), and high SDI (≥0·80).^22^, ^23^, ^24^
Age‐standardized rate (per 100.000)	Comparisons of crude age‐specific rates over time and between populations may be very misleading if the underlying age composition differs in the populations being compared.^25^	$\mathrm{ASR}=\frac{\sum_{i=1}^{A}a_{i}w_{i}}{\sum_{i=1}^{A}w_{i}}\times 100,000$ where *a*_*i*_ and *w*_*i*_ represent the age‐specific rates and the number of persons (or weight) in the same age subgroup of the chosen reference standard population (where *i* denotes the *i*th age class), respectively.^25^

## 3 RESULTS

### 3.1 The global incidence rate of cervical cancer

In 2019, a total of 565 541 new cases of cervical cancer with a confidence level (636435–481 524) were reported in women worldwide, with an incidence of ASR of 13.35 cases per 100 000 people. Statistics show that the lower the SDI index is, the higher age‐standardized incidence rates of cervical cancer will be so that the highest standardized age incidence can be found in countries with low SDI and the lowest in countries with high SDI. This rate is equal to 8.91 in high SDI countries and 23.21 in low SDI countries. According to the World Bank classification, the incidence of ASR has the lowest value (9.21) in the high‐income countries and the highest value (30.29) in the low‐income countries. Among the continents, the highest incidence of ASR is in Africa (24.02) and the lowest is in Europe (10.79). According to the World Health Organization (WHO), the highest standard incidence is in the African region and the lowest is in the Eastern Mediterranean region.

Also, in general and based on GBD regions, the highest incidence of ASR is in Sub‐Saharan African countries (WB) and then Latin America & Caribbean countries.

The highest standardized incidence of cervical cancer has been reported to be in Kiribati (108.8), Palau (66.58), Solomon Islands (57), Guinea (53.61), Lesotho (52.77), Zimbabwe (48.95), Botswana (47.63), Eritrea (44.96), Guinea‐Bissau (44.77) and Haiti (44.12).

However, the lowest standardized incidence of cervical cancer has been reported to be in Egypt (2.84), Syrian Arab Republic (3.25), Kuwait (3.62), Iran (3.99), Jordan (4.03), Iraq (4.61), Palestine (4.66), Turkey (4.67), Malta (4.94) and Saudi Arabia (4.95) (Table 1).

### 3.2 The global mortality rate of cervical cancer

In 2019, a total of 280 479 new deaths due to cervical cancer with the conference level (238864_313930) were reported among women in the world, with deaths ASR per 10^5^ equal to 6.51 per 100 000 people. Statistics show that the lower the SDI index is, the higher the standardized age‐related death rate from cervical cancer will be so the highest deaths ASR is in countries with low SDI and the lowest is in countries with high SDI. This rate is equal to 2.90 in high SDI countries and 15.05 in low SDI countries. According to the World Bank classification, the death ASR has the lowest value in the high‐income group (3.55) and the highest value in the low‐income group (19.59). Among the continents, the highest ASR death is in Africa (15.49) and the lowest in Europe (4.02). According to the World Health Organization, the highest standard incidence is in the African region and the lowest in the European region.

Also in general and based on GBD regions, the highest death ASR is related to Sub‐Saharan Africa.

The highest standardized deaths rate from cervical cancer has been reported in Kiribati (69.52), Guinea (36.16), Lesotho (35.96), Zimbabwe (31.39), Somalia (30.99), Eritrea (30.26), Palau (29.79), Solomon Islands (29.44), Central African Republic (29.31), and Guinea‐Bissau (29.28).

Meanwhile, the lowest standardized death rate from cervical cancer has been reported in Kuwait (1.76), Egypt (1.77), Syrian Arab Republic (1.78), Finland (1.78), Malta (1.78), Iceland (1.9), Luxembourg (1.94), Jordan (2.04), Iran (2.06) and Australia (2.14) (Table 2).

**TABLE 2:** Cervical cancer incidence cases, age‐standardized incidence rate, deaths, age‐standardized mortality rate, DALYs, age‐standardized DALY rates, YLLs, age‐standardized YLLs rates, YLDs, and age‐standardized YLDs rates in 2019

	Incidence cases	Incidence ASR per 10^5^	Deaths cases	Deaths ASR per 10^5^	DALYs number	DALYs ASR per 10^5^	YLLs number	YLLs ASR per 10^5^	YLDs number	YLDs ASR per 10^5^
	(95% CI)	(95% CI)	(95% CI)	(95% CI)	(95% CI)	(95% CI)	(95% CI)	(95% CI)	(95% CI)	(95% CI)
Global	565 541	13.35	280 479	6.51	8 955 013	210.64	8 712 962	204.89	242 051	5.75
	(481 524_636 435)	(11.37_15.03)	(238864_313930)	(5.55_7.29)	(7547733_9978462)	(177.67_234.85)	(7365279_9728886)	(173.07_228.86)	(171644_326024)	(4.07_7.75)
SDI										
High SDI	63 864	8.91	26 173	2.90	672 113	89.72	641 596	85.26	30 517	4.46
	(55710_71455)	(7.74_9.99)	(22823_28149)	(2.6_3.1)	(608748_721998)	(81.88_95.85)	(580762_684914)	(78.45_90.62)	(21168_41571)	(3.08_6.08)
High‐middle SDI	113 123	11.59	51 771	4.89	1 543 704	154.69	1 492 922	149.36	50 782	5.32
	(89780_129153)	(9.18_13.24)	(41664_57874)	(3.92_5.47)	(1235997_1729870)	(124.02_173.51)	(1191878_1676854)	(119.06_167.64)	(34921_69817)	(3.66_7.34)
Low SDI	78 821	23.21	45 540	15.05	1 632 490	477.53	1 602 067	469.09	30 423	8.43
	(61613_97925)	(18.31_28.76)	(35797_56258)	(11.92_18.46)	(1271609_2044290)	(374.33_591.38)	(1248560_2008867)	(367.54_580.44)	(20152_43752)	(5.73_11.99)
Low‐middle SDI	125 963	15.78	66 678	8.85	2 282 245	285.64	2 230 841	279.35	51 404	6.29
	(107883_150105)	(13.57_18.87)	(57270_81245)	(7.62_10.83)	(1948327_2722926)	(244.64_342.16)	(1902939_2671047)	(239_335.78)	(36161_69166)	(4.45_8.42)
Middle SDI	183 337	13.44	90 100	6.78	2 817 246	204.60	2 738 503	198.89	78 743	5.72
	(144492_208859)	(10.61_15.28)	(71333_103200)	(5.4_7.76)	(2223191_3217721)	(161.92_233.49)	(2157802_3126816)	(157.36_226.82)	(55202_106370)	(4.02_7.71)
World bank income level										
World bank high income	79 094	9.21	33 190	3.05	846 454	94.36	808 967	89.77	37 487	4.58
	(68439_88835)	(7.98_10.35)	(29017_35666)	(2.72_3.27)	(750866_906426)	(85.22_100.75)	(722369_866196)	(81.68_95.78)	(26094_50860)	(3.16_6.21)
World bank low income	64 322	30.29	37 256	19.59	1 325 105	619.77	1 300 397	608.79	24 708	10.97
	(47789_80697)	(22.79_37.79)	(28500_46704)	(15.18_24.31)	(996969_1670798)	(471.6_778.85)	(979930_1641838)	(463.3_764.56)	(15775_35941)	(7.18_15.83)
World bank lower middle income	190 582	13.57	100 125	7.62	3 404 838	241.58	3 326 372	236.15	78 466	5.43
	(163555_230583)	(11.66_16.39)	(84549_127255)	(6.47_9.79)	(2879036_4266168)	(204.91_303.5)	(2786056_4162231)	(198.89_297.37)	(54975_108816)	(3.8_7.5)
World bank upper middle income	231 109	13.49	109 690	6.19	3 371 381	193.10	3 270 174	187.13	101 207	5.97
	(173791_267544)	(10.15_15.62)	(83717_126842)	(4.73_7.15)	(2536703_3905565)	(145.5_224.25)	(2462855_3797624)	(141.14_217.21)	(67261_139387)	(3.98_8.24)
Continents										
Africa	100 882	24.02	57 328	15.49	2 013 205	475.55	1 973 860	466.76	39 345	8.78
	(78274_123781)	(18.79_29.13)	(44735_69567)	(12.27_18.62)	(1554998_2473422)	(367.89_578.31)	(1522866_2426697)	(361.6_568.31)	(26276_55832)	(5.92_12.42)
America	99 344	16.37	45 880	7.06	1 412 411	230.93	1 368 848	223.60	43 563	7.33
	(86452_113504)	(14.22_18.73)	(41564_50779)	(6.38_7.83)	(1274478_1573926)	(208.08_257.41)	(1234552_1524455)	(201.29_249.34)	(30364_58147)	(5.12_9.81)
Asia	297 402	11.70	146 502	5.79	4 693 918	183.34	4 565 684	178.30	128 234	5.04
	(238273_343878)	(9.38_13.51)	(118910_170830)	(4.7_6.75)	(3779579_5446237)	(147.71_212.54)	(3682644_5330317)	(143.9_208.16)	(88980_176159)	(3.5_6.92)
Europe	67 160	10.79	30 408	4.02	824 336	128.22	793 756	123.06	30 580	5.16
	(57710_76474)	(9.18_12.3)	(27094_33558)	(3.56_4.44)	(726198_913992)	(112.16_142.62)	(703004_877841)	(108.17_136.65)	(21266_42064)	(3.54_7.13)
WHO regions										
African region	93 772	27.87	53 396	18.08	1 878 932	554.98	1 842 470	544.85	36 462	10.13
	(72680_114333)	(21.87_33.89)	(41571_64892)	(14.26_21.76)	(1455936_2310125)	(430.28_676.85)	(1427759_2266402)	(422.64_666.12)	(24021_51877)	(6.78_14.33)
Eastern mediterranean region	18 394	6.92	9444	4.05	331 307	124.18	323 532	121.40	7775	2.78
	(14422_22642)	(5.52_8.43)	(7456_11530)	(3.21_4.89)	(254792_407536)	(97.83_151.98)	(249106_399272)	(95.71_148.6)	(5193_10769)	(1.88_3.82)
European region	73 345	11.09	33 081	4.20	918 838	135.24	885 494	129.96	33 343	5.27
	(63335_83205)	(9.48_12.62)	(29639_36531)	(3.74_4.64)	(811157_1016627)	(118.79_150.01)	(787659_978687)	(114.8_144.07)	(23124_45816)	(3.63_7.28)
Region of the Americas	99 344	16.37	45 880	7.06	1 412 411	230.93	1 368 848	223.60	43 563	7.33
	(86452_113504)	(14.22_18.73)	(41564_50779)	(6.38_7.83)	(1274478_1573926)	(208.08_257.41)	(1234552_1524455)	(201.29_249.34)	(30364_58147)	(5.12_9.81)
South‐East Asia region	128 159	13.05	66 891	7.16	2 258 636	229.21	2 205 734	223.93	52 902	5.28
	(106048_159948)	(10.82_16.24)	(54920_89489)	(5.89_9.61)	(1842723_2972147)	(187.06_302.5)	(1792983_2909564)	(182.66_295.61)	(36466_74683)	(3.66_7.43)
Western pacific region	151 207	11.49	71 137	5.09	2 135 723	158.17	2 068 297	152.95	67 426	5.22
	(94922_184177)	(7.21_13.98)	(45603_86835)	(3.26_6.22)	(1333747_2632365)	(98.92_194.82)	(1280171_2561814)	(95.23_189.43)	(40108_95176)	(3.1_7.35)
GBD region										
East Asia & pacific ‐ WB	184 472	11.83	87 428	5.37	2 664 506	167.53	2 582 763	162.22	81 743	5.31
	(130835_218161)	(8.38_14)	(63621_103533)	(3.91_6.36)	(1901459_3159034)	(119.67_198.41)	(1837004_3077005)	(115.26_193.2)	(52835_112937)	(3.41_7.36)
East Asia	115 377	11.17	55 960	5.18	1 696 322	159.12	1 645 532	154.13	50 790	4.99
	(64346_147115)	(6.25_14.26)	(33187_71362)	(3.09_6.59)	(972552_2166628)	(91.62_202.95)	(943862_2116370)	(88.81_198.08)	(27021_72628)	(2.68_7.15)
Oceania	1329	28.22	669	16.41	24 914	521.37	24 369	510.52	545	10.85
	(860_1820)	(19_38.09)	(448_913)	(11.5_22.19)	(16093_34055)	(347.49_709.69)	(15758_33277)	(340.22_696.79)	(315_806)	(6.52_15.84)
Southeast asia	52 062	14.48	25 129	7.36	808 250	223.36	785 702	217.21	22 549	6.15
	(41929_68668)	(11.73_19)	(20525_34980)	(6.03_10.33)	(653206_1088292)	(181.44_302.65)	(635849_1061822)	(175.91_295.38)	(15542_32697)	(4.24_8.88)
Sub‐Saharan Africa ‐ WB	94 649	28.43	54 345	18.63	1 916 111	571.47	1 879 491	561.21	36 620	10.26
	(73427_115522)	(22.35_34.58)	(42207_66192)	(14.75_22.43)	(1476352_2359741)	(442.8_696.7)	(1446384_2317841)	(435.37_686.35)	(24078_51981)	(6.86_14.48)
Central Sub‐Saharan Africa	12 297	32.32	7296	21.67	261 630	678.72	256 989	667.30	4641	11.42
	(8233_16878)	(21.74_44.74)	(4908_10058)	(14.49_30.24)	(176038_360427)	(454.78_932.08)	(173155_354176)	(447.67_916.42)	(2785_7115)	(6.75_17.64)
Eastern Sub‐Saharan Africa	36 335	31.79	21 112	21.13	758 613	660.28	744 599	648.86	14 014	11.41
	(25756_48449)	(22.9_41.68)	(15477_27856)	(15.15_27.62)	(557094_1022325)	(484.65_874.17)	(546695_998718)	(476.27_855.79)	(8874_21323)	(7.32_17.05)
Southern Sub‐Saharan Africa	12 021	32.90	6561	19.34	213 941	586.79	209 239	574.34	4703	12.45
	(9740_14445)	(26.88_39.48)	(5390_7752)	(15.82_22.77)	(173968_254952)	(476.2_698.37)	(170773_249771)	(467.51_684.79)	(3155_6417)	(8.44_16.94)
Western Sub‐Saharan Africa	33 374	25.47	19 088	16.83	672 604	507.97	659 617	498.80	12 986	9.17
	(26137_42535)	(20.17_31.94)	(15041_24011)	(13.38_21)	(524740_854863)	(398.99_640.76)	(515399_837630)	(391.38_629.04)	(8442_18342)	(6.01_12.89)
South Asia ‐ WB	102 182	12.28	54 417	6.95	1 870 822	224.63	1 829 184	219.75	41 638	4.88
	(82027_127652)	(9.88_15.35)	(43874_70911)	(5.63_9.09)	(1501585_2406309)	(180.35_289.73)	(1460972_2367947)	(176.47_285.16)	(28312_59353)	(3.35_6.95)
South Asia	100 020	12.37	53 303	7.01	1 833 690	226.59	1 792 969	221.69	40 720	4.91
	(80106_124770)	(9.94_15.46)	(42871_69946)	(5.66_9.21)	(1466658_2370915)	(181.92_292.64)	(1430672_2320477)	(177.38_288.19)	(27698_58249)	(3.36_7)
Latin America & Caribbean ‐ WB	77 788	21.44	37 215	10.24	1 170 047	321.75	1 136 781	312.58	33 266	9.17
	(67060_90914)	(18.49_25.06)	(33067_42108)	(9.11_11.59)	(1036718_1329375)	(285.06_365.56)	(1006810_1291559)	(276.95_355.16)	(23234_44902)	(6.4_12.37)
Andean Latin America	9100	29.74	4278	14.37	129 594	422.28	125 658	409.55	3936	12.73
	(6929_11615)	(22.67_37.83)	(3317_5382)	(11.18_18.04)	(99418_165403)	(323.97_538.4)	(95964_161070)	(313.41_524.4)	(2515_5670)	(8.13_18.27)
Caribbean	6862	26.23	3470	12.95	114 714	438.19	111 866	427.21	2848	10.98
	(5357_8500)	(20.41_32.58)	(2724_4261)	(10.11_15.96)	(86803_145019)	(328.34_557.85)	(84364_141445)	(320.73_543.71)	(1936_3987)	(7.4_15.38)
Central Latin America	28 479	21.45	13 831	10.65	436 918	328.59	424 842	319.58	12 076	9.01
	(23109_35027)	(17.44_26.37)	(11534_16804)	(8.91_12.92)	(361747_538523)	(272.54_404.33)	(349075_522556)	(262.86_392.67)	(8276_16735)	(6.19_12.45)
Tropical Latin America	23 740	17.91	11 580	8.69	365 275	274.27	355 154	266.63	10 121	7.64
	(22128_27179)	(16.69_20.43)	(10715_13657)	(8.04_10.23)	(340281_419750)	(255.5_314.34)	(330359_407919)	(247.94_305.43)	(7238_13607)	(5.47_10.27)
Middle East & North Africa ‐ WB	11 178	5.82	5133	3.09	165 502	87.43	160 510	84.96	4991.796837	2.48
	(8556_13795)	(4.48_7.14)	(4010_6225)	(2.45_3.72)	(125126_204684)	(66.72_106.87)	(122254_198577)	(64.96_103.23)	(3288_6985)	(1.63_3.43)
North Africa and Middle East	14 626	5.78	7005	3.15	221 931	88.28	215 513	85.86	6418.171729	2.42
	(11139_17632)	(4.43_6.89)	(5443_8311)	(2.47_3.69)	(169203_268187)	(68.42_105.74)	(164084_260919)	(66.46_102.71)	(4270_8878)	(1.62_3.32)
Europe & Central Asia–WB	72 777	11.12	32 829	4.21	911 990	135.67	878 906	130.39	33 084	5.29
	(62830_82443)	(9.51_12.66)	(29400_36249)	(3.75_4.65)	(805241_1009126)	(119.19_150.47)	(781936_971761)	(115.3_144.48)	(22927_45411)	(3.65_7.31)
Central Asia	7666	16.00	3423	7.58	119 723	249.41	116 339	242.49	3384	6.92
	(6647_8830)	(13.94_18.4)	(3000_3927)	(6.68_8.7)	(103947_138538)	(217.42_288.15)	(100699_134336)	(211.62_279.81)	(2327_4698)	(4.79_9.55)
Central Europe	13 677	15.80	6883	6.65	190 256	212.08	184 395	204.89	5860	7.19
	(11258_15896)	(12.97_18.48)	(5824_7988)	(5.59_7.75)	(159630_221330)	(177.26_247.37)	(154486_215430)	(171.34_239.84)	(4014_8099)	(4.89_10.04)
Eastern Europe	22 997	14.76	10 037	5.54	308 610	192.88	298 182	185.91	10 428	6.97
	(18912_28032)	(11.91_18.14)	(8472_11910)	(4.62_6.61)	(255790_369569)	(156.89_231.51)	(245103_357718)	(151.15_224.19)	(6881_14703)	(4.54_9.97)
High Income	75 580	9.85	30 855	3.12	798 028	99.38	761 995	94.45	36 033	4.93
	(64076_85474)	(8.31_11.2)	(26706_32981)	(2.74_3.32)	(701275_852180)	(87.08_105.66)	(669621_807226)	(82.83_99.76)	(24714_49014)	(3.34_6.75)
Australasia	1648	8.22	525	2.17	13 577	65.47	12 732	61.16	845	4.31
	(1270_2114)	(6.32_10.59)	(448_583)	(1.88_2.4)	(11844_15056)	(57.37_72.7)	(11156_14085)	(54.3_67.69)	(541_1230)	(2.75_6.37)
Asia Pacific	15 061	10.33	5604	2.70	133 639	85.67	126 160	80.25	7479	5.42
	(11908_17961)	(7.99_12.4)	(4577_6217)	(2.22_2.96)	(109967_146607)	(67.59_93.64)	(104439_137772)	(63.9_87.18)	(4866_10718)	(3.49_7.77)
North America	21 852	8.93	8799	2.99	245 963	96.55	235 530	92.13	10 433	4.42
	(17425_26617)	(7.09_10.93)	(7475_9340)	(2.55_3.15)	(211944_259257)	(83.94_101.88)	(202509_247907)	(80.62_97.03)	(6940_14538)	(2.92_6.2)
Southern Latin America	9844	24.85	4176	9.64	127 490	317.23	123 106	305.88	4384	11.35
	(7273_12855)	(18.23_32.74)	(3552_4598)	(8.17_10.56)	(105401_140054)	(260.3_348.08)	(102486_134687)	(252.97_334.79)	(2739_6401)	(7.05_16.67)
Western Europe	27 174	8.26	11 752	2.65	277 358	79.19	264 467	75.03	12 891	4.16
	(22694_31702)	(6.85_9.68)	(10271_12689)	(2.38_2.85)	(248483_299741)	(71.88_85.34)	(238401_283387)	(68.39_80.38)	(8815_17604)	(2.81_5.75)

### 3.3 The global burden of cervical cancer

In 2019, DALYs due to cervical cancer in women were reported at 8955013 with a 95% confidence level (7547733_9978462), from which 8 712 962 were related to YLLs cases and 242 051 to YLDs cases. Also, the worldwide DALYs ASR was reported at 210.64 and this number for YLLs ASR and YLDs ASR was 204.89 and 5.75 respectively. According to SDI classification, the highest DALYs ASR is in low SDI countries and the lowest is in high SDI countries. The YLLs and YLDs ASR are also the highest in low SDI countries.

According to the World Bank classification, the highest value of DALYs ASR is related to World Bank Low‐income countries and the lowest value is related to high‐income countries. Also in the case of YLLs ASR and YLDs ASR, the highest value is related to low‐income countries and the lowest value is related to high‐income countries.

In different continents, the highest DALYs ASR belongs to the African and then American continents. For the YLLs ASR, the highest value belongs to the African continent and the lowest value belongs to the European continent, but for the YLDs ASR the highest value belongs to the African continent and the lowest value belongs to the Asian continent.

According to the World Health Organization regions, the highest DALYs ASR is in the African region, followed by the American region, and the lowest is in the Eastern Mediterranean and European regions. But regarding YLDs ASR, the highest value is in Africa and the lowest is in the Eastern Mediterranean regions.

Based on GBD regions, the highest standardized age of DALYs, YLLs ASR, and YLDs ASR are in Sub‐Saharan Africa and then Latin America & Caribbean‐WB.

The highest DALYs ASR has been reported in Kiribati (2143.06), Guinea (1143.8), Lesotho (1087.77), Solomon Islands (1018.69), Somalia (1013.76), Eritrea (973.58), Zimbabwe (957.22), Central African Republic (955.25), Guinea‐Bissau (937.63) and Mozambique (915.04).

And the lowest DALYs ASR has also been reported in Kuwait (44.34), Egypt (45.13), Syrian Arab Republic (46.56), Finland (47.49), Malta (50.94), Iran (54.11), Iceland (54.93), Jordan (55.25), Luxembourg (55.34) and Switzerland (58.09).

The highest YLDs ASR has also been reported in Kiribati (39.28), Palau (29.01), Solomon Islands (23.15), Guinea (18.66), Botswana (18.44), Lesotho (17.93), Zimbabwe (17.11), Saint Vincent and the Grenadines (16.95), Sao Tome and Principe (16.65), and Nauru (16.64).

Meanwhile, the lowest YLDs ASR has been observed in Egypt (1.14), Syrian Arab Republic (1.39), Kuwait (1.64), Iran (1.74), Jordan (1.78), Palestine (1.83), Turkey (1.97), Iraq (1.99), Sudan (2.21) and Saudi Arabia (2.23).

The highest YLLs ASR has been reported in Kiribati (2103.78), Guinea (1125.13), Lesotho (1069.85), Somalia (999.57), Solomon Islands (995.55), Eritrea (957.88), Central African Republic (941.77), Guinea‐Bissau (921.96) and Mozambique (899.74).

Meanwhile, the lowest YLLs ASR has been reported in Kuwait (42.7), Egypt (43.99), Finland (44.91), Syrian Arab Republic (45.18), Malta (48.55), Iceland (52.06), Iran (52.38), Jordan (53.47) and Switzerland (55.31) (Table 2).

## 4 DISCUSSION

Despite recent advances in the diagnosis and treatment of cervical cancer, with 280 479 deaths reported in 2019, this cancer was the fifth leading cause of neoplasm death in women worldwide.^26^ Compared to many cancers, the average age of women who develop cervical cancer is low, so the number of years lost due to this disease is significant and can deprive women of survival at the peak of their social and family life.^27^ In 2019, this cancer caused 8.9 million DALYs.

In 2019, a total of 565 541 cases of cervical cancer were reported worldwide, of which only 11% were detected in high SDI countries. The age‐standardized incidence of cervical cancer in the world in 2019 was 13.35 per 100 000 women and the age‐standardized mortality rate was 6.51 per 100 000 women. This rate also varied significantly in different parts of the world. Among the various cancers, the highest variability in cancer incidence and mortality is seen in this malignancy.^28^ According to the results of GBD in 2019, the highest incidence rates of ASR, death ASR, and DALYs ASR are seen in low SDI countries.

Of the 21 GBD regions, the highest age‐standardized incidence, mortality, and DALYs have been reported in Southern and Central Sub‐Saharan Africa, respectively. Numerous factors have changed the rate of cancer in these areas. The high incidence of HPV in the world is also seen in sub‐Saharan Africa.^29^ Women in this region start having sex at a younger age due to cultural norms, followed by a younger pregnancy and higher parity. In addition, financial, socio‐cultural, and governmental barriers have made screening in these areas less effective.^30^, ^31^ The cost of Pap smear test, lack of access or difficult access to service providers and long waiting time are some of the system barriers in this region that indicate their socio‐economic status.^32^ The five‐year survival rate for women with cervical cancer in these regions is 33%, which is much lower than the 80% rate in high‐income countries.^33^ In contrast, GBD (2019) has reported the lowest ASIR of cervical cancer in North Africa and the Middle East. The use of various prevention methods as well as standard treatment algorithms is the main reason for obtaining current statistics. Social norms and religious beliefs, as well as the limitation of sexual activity only in marriage according to Islamic law compared to the Western societies, have led to a decline in such statistics in the Middle East.^6^, ^34^, ^35^

The difference in cervical cancer rate depends on several factors such as the human development index, sexual behaviors, fertility patterns, and the degree of adherence to screening programs. Meanwhile, social differences also play a significant role in the differences in incidence and mortality of cervical cancer. Compared to higher socioeconomic status, lower socioeconomic status increases the risk of cervical cancer by 2–3 times.^36^

Cervical cancer is considered a preventable disease. Screening reduces the mortality of this cancer by identifying and treating precancerous lesions at a lower stage.^37^ Also, performing a single cervical cancer screening test in a lifetime reduces this cancer by 25–36%.^38^ Screening is most effective when women at high risk of precancerous lesions take part in screening programs. Lack of access to quality screening programs is one of the challenges facing many women around the world. On the other hand, in many parts of the world, opportunistic screening is being implemented that does not target high‐risk individuals.^7^ While, in recent years, screening programs and epidemiologic indicators of cervical cancer have been affected by the COVID‐19 crisis.^39^ Also, during this period, receiving appropriate treatment by cervical cancer patients was appealed to challenges due to psychological distress (mostly fear of infectiousness).^40^

In addition to screening, HPV vaccination is an effective way to prevent cervical cancer, which has been used in some areas. Nevertheless, cervical cancer is still one of the most common cancers in women, leading to high morbidity and mortality in many women. From 2008 to 2012, the incidence of cervical cancer in young women decreased in some countries, which can be attributed to the introduction of vaccination in this group.^41^ Although HPV vaccination has been introduced as an approach to reducing the incidence of this disease, its use is insignificant in many areas. The high cost of vaccination is one of the barriers to its widespread use, especially in poor and low‐income countries.^42^

Not all cases of intraepithelial neoplasia progress to invasive cancer. Progression from CIN to invasive cancer requires HPV and some cofactors, such as smoking, high parity, multiple sexual partners, prolonged use of hormonal contraceptives, and failure to perform regular screening programs.^6^ In areas where the prevalence of risk factors is higher, cervical cancer has a higher incidence and case fatality rate.^26^

Cervical cancer is a clear example of inequality in health services in a country and between different countries. Differences in exposure to risk factors and injustice in access to screening, diagnostic, and treatment centers can explain some of these significant differences between different regions of the world.^43^ Inequality in mortality is wider than inequalities in the incidence of cervical cancer. Patient survival and mortality are affected by the stage of cancer at diagnosis, access to health care, and appropriate treatment.^44^

In 2015, cervical cancer was the 18th leading cause of death in high‐SDI countries, while it was 2nd in low‐SDI countries. Compared to developed countries, the mortality rate of cervical cancer in low and middle‐income areas is 18 times higher.^28^ High SDI countries account for a smaller share of incidence, mortality, and DALYs of cervical cancer. This rate has been controlled in many areas with early diagnosis and appropriate treatment.^45^

Implementing cancer control measures around the world requires appropriate action and concerted effort by the governments of each country and the international agencies in the public and private sectors. Therefore, in May 2018, the World Health Organization called for the elimination of cervical cancer as one of the public health problems to reduce the health, social and economic burden of cervical cancer by removing related barriers.^46^ Given that cervical cancer is highly preventable, access to screening services and the presence of trained and knowledgeable health care staff can reduce illness, suffering, and death caused by this malignancy. So, according to the World Health Organization, using a combination of interventions to achieve these goals should be considered.^47^ Therefore, it is recommended to use the national and international potentials to reduce the incidence of this malignancy.

This study had some limitations that should be noted. First, although GBD (2019) has collected data from various sources, data from some areas is limited, which can affect the results of this study. Second, GBD statistics were based on a set of data sources such as cancer registry data and cytological results, while access to these resources is limited in some low‐income countries, and estimating statistics may be erroneous. Third, due to several years delay in presentation of cancer data, up to date data not available.

## AUTHOR CONTRIBUTIONS

**zohreh momenimovahed:** Data curation (equal); investigation (equal); methodology (equal); project administration (equal); supervision (equal); validation (equal); writing – original draft (equal); writing – review and editing (equal). **Afrooz mazidi moradi:** Conceptualization (equal); data curation (equal); formal analysis (equal); methodology (equal); writing – original draft (equal); writing – review and editing (equal). **Parang Maroofi:** Data curation (equal); formal analysis (equal); methodology (equal); writing – original draft (equal); writing – review and editing (equal). **Leila Allahqoli:** Conceptualization (equal); data curation (equal); methodology (equal); project administration (equal); supervision (equal); writing – original draft (equal); writing – review and editing (equal). **Ibrahim Alkatout:** Conceptualization (equal); funding acquisition (equal); investigation (equal); project administration (equal); resources (equal); supervision (equal); validation (equal); writing – original draft (equal); writing – review and editing (equal). **Hamid Salehiniya:** Conceptualization (equal); data curation (equal); formal analysis (equal); methodology (equal); project administration (equal); validation (equal); visualization (equal); writing – original draft (equal); writing – review and editing (equal).

## CONFLICT OF INTEREST

The authors declare no conflict of interest.

## ETHICS STATEMENT

The study was approved by the ethics committee of the Birjand University of Medical Sciences (ethics committee approval code IR.BUMS.REC.1400.316). As we used routinely collected anonymized electronic data, patient consent was not required.

## Data Availability

Data sharing is not applicable to this article as no new data were created or analyzed in this study.
